# Anti-inflammatory properties of extracts from *Chimonanthus nitens* Oliv. leaf

**DOI:** 10.1371/journal.pone.0181094

**Published:** 2017-07-10

**Authors:** Qi Sun, Jiajin Zhu, Feiwei Cao, Fengjia Chen

**Affiliations:** Zhejiang Key Laboratory for Agro-Food Processing, Zhejiang Engineering Center for Food Technology and Equipment, Fuli Institute for Food Science, College of Biosystems Engineering and Food Science, Zhejiang University, Hangzhou, Zhejiang, China; University of British Columbia, CANADA

## Abstract

*Chimonanthus nitens* Oliv. (CN) is a species in the family Calycanthaceae. Its leaf is widely used to make traditional herbal tea in southern China and has a wide range of therapeutic effects. The profile of the ethanol extracts from CN leaves was identified by UPLC-QTOF-MS/MS. Forty seven compounds were determined including organic acids, phenolic acids and derivatives, flavonoids, coumarins, fatty acids and other compounds. The effect of the CN extracts on the inflammatory damage in zebrafish and in RAW 264.7 cells was investigated. The extracts demonstrated a strong ability to inhibit the recruitment of neutrophils in LPS-stimulated zebrafish, but macrophage migration was not significantly affected. Pro-inflammatory cytokines (i.e., TNF-α, IL-6 and IL-1β) were also determined by q-PCR. The extracts strongly reduced mRNA expression of TNF-α, IL-6 but not IL-1β in zebrafish model, while significantly inhibited the production of the factors in the RAW 264.7 cells. Therefore, our results suggest that the ethanol extracts of CN leaves may serve as a source of nutraceutical compounds with anti-inflammatory properties.

## Introduction

Inflammation is the response of the organism to tissue injury caused by chemicals, bacteria, trauma, or other harmful agents. Triggered by a range of stimuli including damaged cells, pathogens and cytokines such as interleukin-6 (IL-6), IL-1β, and tumor necrosis factor alpha (TNF-α), granulocytes (such as neutrophils) and monocytes, which can then differentiate into macrophages, are attracted to the damaged tissues through chemotaxis, amplify inflammatory reactions and initiate phagocytosis [1]. Inflammation is characterized by swelling and pain and also an important factor involved in the development of many human chronic diseases including cardiovascular disease, diabetes, neurodegenerative diseases and even cancer [2–5]. Nowadays, many natural products, such as flavonoid glycosides, steroids, alkaloids and phenolic glycosides, show potential anti-inflammatory ability [6, 7]. For example, suffruticosol A, a phenolic compound from *Paeonia lactiflora* Pall. Seedcases, may mediate airway inflammation in mice [8]. Additionally, anthocyanin-rich black mulberry extracts significantly reduced pro-inflammatory cytokines in the serum of ear edema mice [9].

The genus *Chimonanthus* (wintersweets) is endemic to China, which includes six species. *Chimonanthus nitens* Oliv. (CN), commonly known as “Liang Ye La Mei” in China, an evergreen shrub, is a member of the family Calycanthaceae. Traditionally, the leaves of CN are widely used to treat common cold and influenza, which are related to its anti-inflammatory property [10, 11]. In the southern China, *Chimonanthus* leaves are also made into functional herbal tea known as “Shi Liang Cha/Shan La Mei Cha” [10, 12]. In addition, the leaves of CN are promising candidates for dietary interventions targeting obesity and inflammatory bowel disease [11, 13].

The genus *Chimonanthus* has been reported to contain alkaloids, flavonoids, coumarins, terpenes and steroids [13–16]. However, most of the research mainly focused on *Chimonanthus praecox* (L.) Link and *Chimonanthus nitens var*. *salicifolius* (S.Y.Hu) H.D.Zhang rather than *Chimonanthus nitens* Oliv. The full profiles of phytochemicals of CN leaves have not been reported, in which may have a wide range of potential anti-inflammatory drugs. Moreover, there has been little work focused on the functional analysis of CN leaf extracts in animal or cell inflammation models.

Zebrafish (*Danio rerio*) is a useful scientific model organism for studies of vertebrate development and gene function [17]. Transgenic zebrafish with organs or cells labeled by fluorescent proteins represent a less labor-intensive way of visualizing organs or cells in the living organism [18]. Migration of macrophages and neutrophils have been reported to measure the severity of inflammation in zebrafish [19–21]. Furthermore, murine RAW 264.7 macrophage model is one of the typical lipopolysaccharide (LPS)-induced inflammation models *ex vivo*, and also have been used to evaluate the anti-inflammatory ability of the extracts or compounds [22–24].

The objective of this work was to investigate the chemical composition and anti-inflammatory capacity of ethanol extracts from the CN leaves. The profiles of extracts were analyzed by UPLC-QTOF-MS/MS and HPLC-DAD. The ability of extracts of CN leaves to modulate the inflammatory response was studied in LPS-stimulated zebrafish. The number of macrophages and neutrophils migrating to the damaged tissues was counted. The mRNA expression of inflammatory cytokines (IL-6, IL-1β and TNF-α) was measured by quantitative real-time PCR. The expression of pro-inflammatory cytokine mediators in LPS-stimulated zebrafish and cell models was examined by enzyme linked immunosorbent assay (ELISA).

## Material and methods

### 2.1. Plant materials

The leaves of CN were collected in August 2015 from plants cultivated in Huanggang village (Chishi Township, Yunhe County, Lishui, Zhejiang province, China, 28° 08′ 04″ N, 119° 26′ 35″ E). The botanical identification was provided by Dr. Chaodong Shen (Landscape Architecture Institute, Zhejiang University) and Dr. Chengxin Fu (Institute of Plant Biology, Zhejiang University). A voucher specimen (No. 2017001) was deposited in the Institute of Plant Biology, College of Life Sciences, Zhejiang University, Hangzhou, China, after the identification of species. Yunhe County Bureau of Agriculture issued the permission to collect plant material at this location. The fresh leaves were dried in an oven at 40°C for 12 h and then powdered and stored at 4°C until extraction and analysis.

### 2.2. Chemicals

Analytical grade methanol and ethanol were purchased from Sinopharm Chemical Reagent Co. (Shanghai, China). Dimethylsulphoxide (DMSO), ethyl 3-aminobenzoate methanesulfonate (Tricaine), dexamethasone (DEX), LPS, 3-(4,5-dimethylthiazol-2-yl)-2,5-diphenyltetrazolium bromide (MTT) and phenol red were purchased from Sigma-Aldrich Co. (St. Louis, MO, United States). PBS (1×) was purchased from Keyi Biotechnology Co. (Hangzhou, Zhejiang, China). HPLC grade acetonitrile, methanol, formic acid, phosphoric acid, and analytical standards of quercetin (≥98.5%), kaempferol (≥98.0%), kaempferol 3-O-β-rutinoside (≥98.0%) and scopoletin (≥98.0%) were purchased from Aladdin (Shanghai, China). RNAiso Plus, PrimeScript^™^ RT reagent Kit with gDNA Eraser (Perfect Real Time) and SYBR^®^ Premix Ex Taq^™^ (Tli RNaseH Plus) were purchased from TAKARA Biotechnology Co. (Dalian, Liaoning, China). Dulbecco’s modified eagle medium (DMEM) was purchased from HyClone Co. (Logan, Utah, United States). 3,3′,5,5′-Tetramethylbenzidine (TMB) was purchased from Huaan Biotechnology Co. (Hangzhou, Zhejiang, China). Fetal bovine serum (FBS) was purchased from Gibco, ThermoFisher Scientific (Shanghai, China). Penicillin-streptomycin was purchased from Solarbio (Beijing, China). ELISA kits for mouse TNF-α, IL-6 and IL-1β were purchased from X-Y Biotechnology Co. (Shanghai, China).

### 2.3. Sample preparation

Extracts were prepared by suspending CN leaves powders in 1:50 (w/v) of ethanol. The suspensions were shaken for 60 min at 40°C. After filtration, the ethanol extract was concentrated using RV10 rotary evaporator (IKA WORKS, Guangzhou, China) at 40°C under reduced pressure to obtain the dry extract (18 g/100 g leaves powder). For component analysis, the dry extracts were dissolved in methanol (2 g dry leaves powder / 50 mL extracts in methanol). For the animal or cell experiments, the dry extracts were dissolved in DMSO (1 g dry extracts / 20 mL DMSO).

### 2.4. HPLC-DAD analysis and UPLC-QTOF-MS/MS analysis

Typical compounds were evaluated by HPLC (Shimadzu Scientific Instruments, Kyoto, Japan). Detection and quantification were carried out with LC-20AD pumps, a SPD-M20A Diode Array Detector (DAD), a CTO-20A column oven, a DGU-20A_3R_ degasser and SIL-20A auto sampler (Shimadzu Scientific Instruments, Kyoto, Japan). Separations were conducted at 40°C on InertSustain C_18_ column (5 μm, 250 × 4.6 mm I.D) (Shimadzu Scientific Instruments, Kyoto, Japan). The flow rate was 1 mL/min, and the absorbance was detected at 360 nm. The mobile phases were water with phosphoric acid (0.1%) (phase A) and methanol (phase B), and the solvent gradient changed according to the following conditions: 0.1 min, 20% B; 5 min, 20% B; 15 min, 25% B; 25 min, 35% B; 35 min, 45% B; 45 min, 55% B; 55 min, 80% B; 65 min, 20% B. The injection volume was 10 μL. Scopoletin, kaempferol-3-O-β-D-rutinoside, quercetin, kaempferol were used as standards. Identification and quantitative analysis were conducted by comparison with standards. The amount of compounds was expressed as mg/g of dry CN leaves using external calibration curves, which were obtained for each standard.

UPLC-QTOF-MS/MS analysis of the CN extracts was performed by the same column at 30°C on a Waters UPLC system (Waters Corp., Milford, MA, United States) coupled to an AB TripleTOF 5600^+^ System (AB SCIEX, Framingham, United States). The absorbance was detected at 360 nm. The injection volume was 5 μL. The gradient eluent, at flow rate of 1 mL/min, and the mobile phases were water with formic acid (0.1%) as solvent system A and acetonitrile as solvent system B, and the solvent gradient changed according to the following conditions: 0 min, 6% B; 20 min, 20% B; 30 min, 25% B; 40 min, 45% B; 50 min, 80% B; 60 min, 95% B. Analysis parameters were set using a negative-ion mode with a scan range from *m/z* 100 to 1500. Source voltage was −4.5 kV and the source temperature was 550°C. The pressure of Gas 1 (Air) and Gas 2 (Air) were set to 50 psi. The pressure of Curtain Gas (N_2_) was set to 30 psi. Maximum allowed error was set to ± 5 ppm. Declustering potential (DP) was 100 V. Collision energy (CE) was 10 V. For MS/MS acquisition mode, the parameters were almost the same except that the collision energy (CE) was set at 40 ± 20 V. Ion release delay (IRD) was at 67. Ion release width (IRW) was at 25. The exact mass calibration was performed automatically using the Automated Calibration Delivery System before each analysis.

### 2.5. Total flavonoid content analysis

The total flavonoid content was measured using method described previously [25] with a slight modification. The total flavonoid content was expressed as equivalents of rutin [26], instead of catechin [25]. Briefly, extract was mixed with 0.3 mL 5% NaNO_2_ solution for 6 min followed by addition of 0.3 mL 10% Al(NO_3_)_3_ solution for 6 min. 4 mL of 1 M NaOH solution was then added to the reaction mixture. Absorbance was determined at 510 nm using rutin as standard.

### 2.6. Zebrafish maintenance and embryo collection

All animal procedures were performed in full accordance to the requirement by “Regulation for the Use of Experimental Animals in Zhejiang Province”. This work is approved by the Animal Ethics Committee in the School of Medicine, Zhejiang University (Ethics Code Permit No. ZJU2015-8-26-004Y, issued by the Animal Ethics Committee in the School of Medicine).

Zebrafish (*Danio rerio*) were raised and maintained in the standard Zebrafish Unit (produced by Aisheng Zebrafish Facility Manufacturer Company, Beijing, China) at Zhejiang University under a constant 14 h on/10 h off light cycle at 28°C. Wild type AB strain and *Tg(coro1a*:*GFP; lyz*:*DsRed)* transgenic fish line [27] were obtained from the Key Laboratory for Molecular Animal Nutrition, Ministry of Education, College of Animal Sciences, Zhejiang University (Hangzhou, China) and were used in this study. After natural spawning, fertilized embryos were collected and cultured in dishes in egg water (culture water for young fish and embryo, 0.05% Sea salts, w/v and 0.002% methylene blue, w/v) at 28°C. For *Tg(coro1a*:*GFP; lyz*:*DsRed)* transgenic line, 0.003% (w/v) 1-phenyl-2-thiourea (PTU) was added to the egg water after 12 h to inhibit melanin formation. Three days post fertilization (dpf) zebrafish larvae were crushed in RNAiso Plus for q-PCR analysis. Anesthetic (0.03% tricaine) is used in microinjection to ameliorate suffering. Animals which were not used for q-PCR were sacrificed by using an excess of tricaine.

### 2.7. LPS-induced inflammation assay

Capillary (Sutter Instrument, Cat. No. BF100-58-10) was heated in the middle and pulled into two capillary tips by Micropipette Puller (P-97 Flaming/Brown Micropipette Puller, Sutter Instrument, Novato, CA, United States). Tips were cut appropriately by using sterile blade before microinjection. LPS solution (dissolved in PBS, phenol red was also added to prevent repeated injections) was filled into the tip by using a special pipette tip (Microloader^™^, Eppendorf, Cat. No. 5242956003). 3 dpf larvae were used in this study. The larvae were anesthetized using 0.03% tricaine, then the microinjection was performed with a PLI-100 Microinjection Systems (Harvard Apparatus, Inc., Massachusetts, MA, United States) under a SZX7 Stereo Microscope (Olympus, Japan). After microinjection, the larvae were cultured at 28°C in 6-well culture plates.

### 2.8. Survival analysis

All zebrafish larvae were divided randomly. Part of 3 dpf larvae were cultured in different concentrations of the CN extracts water (egg water; 0.1% DMSO; 5 μg/mL, 10 μg/mL, 25 μg/mL CN extract dissolved in egg water). Another part of 3 dpf larvae were microinjected with 1 nL of different concentrations of LPS (0 mg/mL, 0.1 mg/mL, 0.5 mg/mL, 1 mg/mL, 2 mg/mL, 5 mg/mL LPS dissolved in PBS) into yolk and then were cultured in egg water. The last part of 3 dpf larvae were microinjected with 1 nL of 5 mg/mL LPS (dissolved in PBS, w/v) into yolk and then were cultured in different concentrations of the CN extract water (0.1% DMSO; 5 μg/mL, 10 μg/mL, 20 μg/mL CN extract dissolved in egg water). Treatments were performed in triplicate (33 larvae per group per dose in 10 mL culture water). The larvae whose heartbeats cannot be observed under stereo microscope are considered as dead individuals. Death count was carried out at 6 h post injection (hpi) and 24 hpi, respectively. The doses leading to low mortality were chosen as the experimental doses.

### 2.9. Cell migration count and image

Three dpf *Tg(coro1a*:*GFP; lyz*:*DsRed)* transgenic line larvae were microinjected with 1 nL of 0.5 mg/mL LPS (dissolved in PBS, w/v) into yolk or 0.5 nL of the same LPS solution into 5–8 somite muscle and then were cultured in different concentrations of the CN extract water (egg water; 5 μg/mL, 10 μg/mL, 20 μg/mL CN extract or 5 μg/mL DEX dissolved in egg water). At 6 hpi, macrophages and neutrophils migration counts were carried out under a SMZ1000 fluorescence stereomicroscope (Nikon, Japan) and the photos were taken under an AZ100 microscope (Nikon, Japan).

### 2.10. q-PCR analysis

Three dpf larvae were microinjected with 1 nL of 5 mg/mL LPS (dissolved in PBS, w/v) into yolk and then were cultured in different concentrations of the CN extract water (egg water; 5 μg/mL, 10 μg/mL CN extract or 5 μg/mL DEX dissolved in egg water). Treatments were performed in triplicate (33 larvae per group per dose in 10 mL culture water). The larvae were collected at 24 hpi. Total RNA from different samples were extracted using RNAiso Plus (Takara) according to manufacturer’s instructions. RNA concentrations and purity were assessed by measuring absorbance ratios at A260/A280 nm using NanoDrop ND-1000 (Thermo Scientific, Wilmington, DE, United States). After that, the RNA of treatments was reverse-transcribed to cDNA with RNA PCR kit (TaKaRa). Q-PCR was performed with SYBR Premix Ex Taq (TaKaRa) on Step One RT-PCR system (Applied Biosystems, Forster, CA, United States) according to the manufacturer’s instructions. Three biological replicates were performed. Relative expression was normalized to the internal control gene β-actin gene with 2^−ΔΔCT^ method. The sequences of the primer pairs used for q-PCR were as follows: β-actin forward primer: ATGGATGAGGAAATCGCTGCC, reverse primer: CTCCCTGATGTCTGGGTCGTC; TNF-α forward primer: ACCAGGCCTTTTCTTCAGGT, reverse primer: TGCCCAGTCTGTCTCCTTCT; il-6 forward primer: TCAACTTCTCCAGCGTGATG, reverse primer: TCTTTCCCTCTTTTCCTCCTG; il-1β forward primer: TGGACTTCGCAGCACAAAATG, reverse primer: CACTTCACGCTCTTGGATGA.

### 2.11. Cell culture and cell viability assay

RAW 264.7 cells were purchased from the Cell Bank of the Chinese Academy of Sciences (Shanghai, China; Cat. No. TCM13). Cells were maintained in DMEM supplemented with 10% FBS and 1% penicillin-streptomycin at 37°C in a 5% CO_2_ humid atmosphere.

The cytotoxicity of CN leaf extract in RAW 264.7 cells was tested by MTT assay. Cells were seeded in 96-well plates at a concentration of 3 × 10^4^ cells/mL and incubated overnight. Thereafter, the culture medium was replaced by serial dilutions of CN leaf extracts (0.1, 0.3, 0.5, 1.0, 1.5, 5, 10, 50 μg/mL) or DEX (0.5 μg/mL) and further incubated for 24 h. 20 μL of the MTT solution (5 mg/mL in PBS) was added to each well, followed by incubation for 4 h in a CO_2_ incubator. After incubation, 150 μL of DMSO was added to each well to dissolve formazan crystals, and optical density was measured at 490 nm using a microplate reader.

### 2.12. Cytokine measurement using ELISA assays

For zebrafish, 3 dpf larvae were treated with the same operations as described previously. Treatments were performed in triplicate (20 larvae per group per dose in 5 mL culture water). Larvae were collected at 24 hpi and put into 50 mL PBS. After homogenization (briefly ultrasonic treatment, 80 kW, 0°C, 30 s), the solution then centrifuged at 12 000 rpm for 15 min at 4°C. The supernatant was collected. Samples and reactive agents were added in the following sequence: 50 μL of TNF-α IgG (Huaan Biotechnology, Hangzhou, China) (1 μg/mL in coating buffer, Na_2_CO_3_ and NaHCO_3_ buffer) were added in each well, and coated overnight at 4°C. Plates were washed three times with 180 μL PBST (0.05% Tween-20 in PBS) and then blocked with 60 μL 1% bovine serum albumin (BSA) of PBST for 1 h at 37°C. Microplates were washed as above and a 50 μL sample diluted 1:50 in PBST with 1% BSA was added and incubated for 1 h at 37°C. BSA was added as negative control. Following a further series of washes (three times for each phase) with PBST, 50 μL of horseradish peroxidase conjugated secondary antibody (Huaan Biotechnology, Hangzhou, China) diluted 1:2000 in PBST with 1% BSA was added and incubated for 45 min at 37°C. After last washing, 100 μL of TMB peroxidase substrate was added to each well and incubated at 37°C in the dark with gentle shaking for 5 min. Reaction was stopped with 90 μL H_2_SO_4_ (2 M) and optical density was read at 450 nm with a microplate reader.

For RAW 264.7 cells, macrophages with CN leaf extracts or DEX (0.5 μg/mL) in the presence of LPS (0.5 μg/mL) were seeded in 6-well plates (at a concentration of 3 × 10^4^ cells/mL) for 24 h. The concentrations of the pro-inflammatory cytokines TNF-α, IL-6, and IL-1β in culture supernatant were determined using ELISA kits following the manufacturer’s protocols.

### 2.13. Statistical analysis

The data are expressed as mean ± S.E. or mean ± SD. One-way ANOVA followed by Fisher's least significant difference (LSD) test was used for assessing significance between the inflammation group and the CN extracts (or DEX) treated groups. Statistical analysis was carried out by using SPSS, version 20 (SPSS Inc., Chicago, United States). Moreover, the UPLC-QTOF-MS/MS data were and analyzed by the software PeakView, version 2.2 and MasterView, version 1.0 (AB SCIEX, Framingham, United States). The MassBank, Pubchem and ChemSpider were applied to analyze the MS^n^ data.

## Results

### 3.1. UPLC-QTOF-MS/MS analysis of ethanol extracts of *Chimonanthus nitens* Oliv. leaves

**Fig 1:**
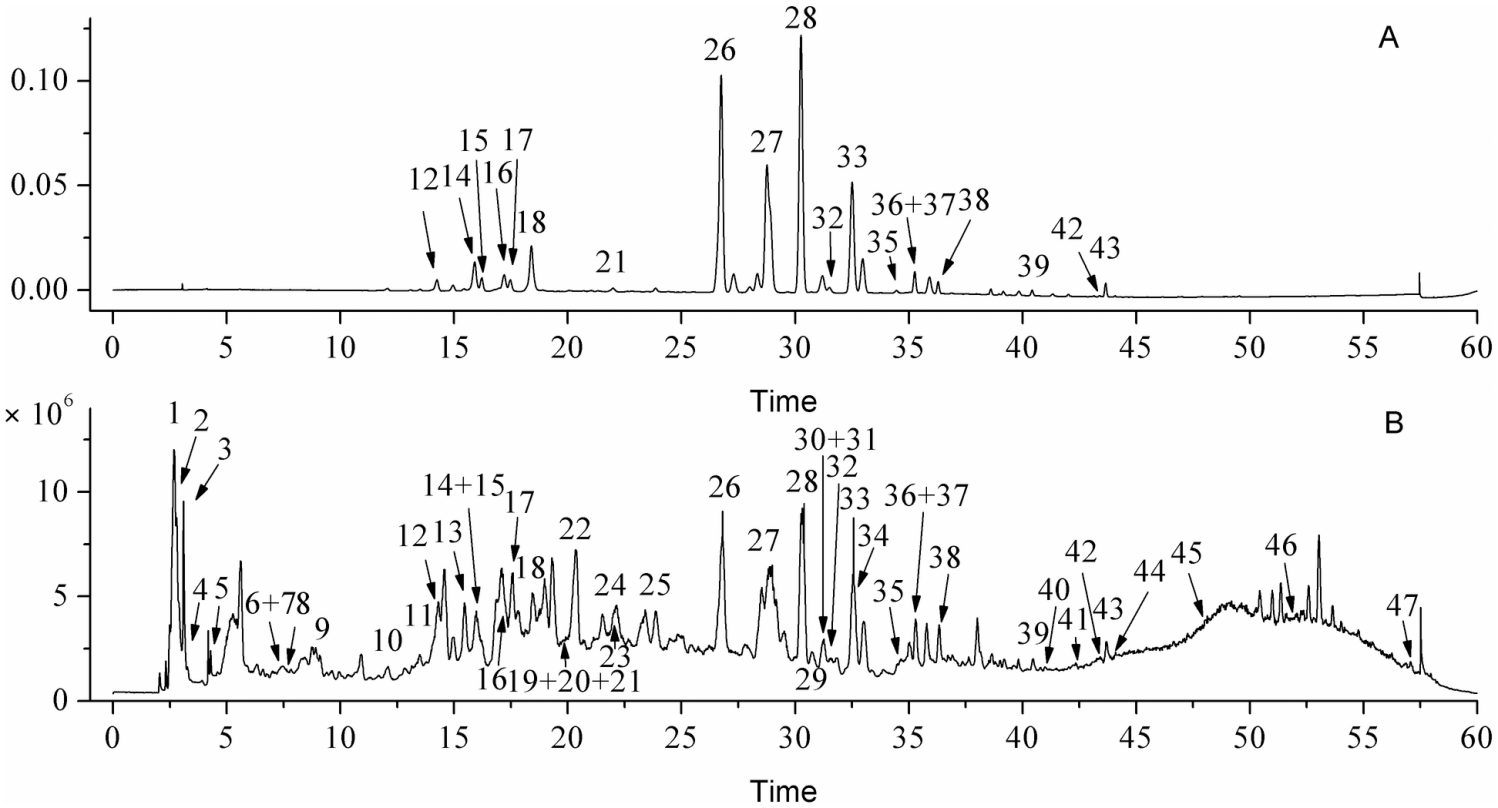
A. DAD chromatogram at 360 nm of *Chimonanthus nitens* Oliv. leaves extracts; B. Total ion chromatogram (TIC) of CN extracts in negative ion mode.

Forty seven chemical compounds were identified from the *Chimonanthus nitens* Oliv. (CN) leaf extracts using UPLC-QTOF-MS/MS as listed in Table 1. All the compounds were identified based on their MS^n^ data and by comparing with MS and MS/MS database and literature. Corresponding standards of scopoletin, kaempferol-3-O-β-D-rutinoside, quercetin and kaempferol were also used to identify the compounds. These compounds can be classified into six groups, including 4 organic acids, 7 phenolic acids and derivatives, 22 flavonoids, 4 coumarins, 4 fatty acids and 6 other compounds. Flavonoids contains 3 flavanols and 19 flavonols. Among these flavonoids, compound **15**, **26**, **27**, **30**, **31**, **32** and **39** were identified as quercetin and its derivatives, and **18**, **28**, **33**, **35**, **36**, **37**, **38** and **43** were identified as kaempferol and its derivatives. The total ion chromatogram (TIC) of CN leaf extracts in the negative ion mode and DAD chromatogram at 360 nm is shown in Fig 1. Efficient separation of compounds in the CN leaf extracts was achieved using the reversed-phase C_18_ column in UPLC. Two predominant compounds in CN leaf extracts were kaempferol-3-O-β-D-rutinoside (**28**) and rutin (quercetin-3-rutinoside, **26**), and these were followed by kaempferol-O-glucoside (**33**), isoquercetin (**27**) and (Epi)catechin (**22**).

**Table 1:** Identification of compounds in *Chimonanthus nitens* leaves Oliv. by UPLC -QTOF-MS/MS.

No.	t_R_ (min)	Formula	[M − H]^−^ (*m/z*)		Error (ppm)	Ion intensity	Major fragment ions (*m/z*)	Identification	References
			Measured	Calculated					
Organic acids									
2	2.81	C_7_H_12_O_6_	191.05607	191.05611	−0.2	1703940	191.0554, 85.0310, 93.0353	Quinic acid	[28, 29]
3	2.95	C_6_H_8_O_7_	191.0199	191.01973	0.9	17853	191.0551, 85.0301, 84.0237, 57.0366	Citric acid	[28, 29]
4	3.17	C_4_H_6_O_5_	133.01455	133.01425	2.3	9260	99.9273, 115.9216, 115.0038	Malic acid	[28, 29]
34	32.82	C_9_H_16_O_4_	187.09774	187.09758	0.8	11543	125.0960, 123.0802, 187.0975	Azelaic acid	[28]
Phenolic acids and derivatives									
6	7.32	C_13_H_16_O_10_	331.06682	331.06707	−0.8	51070	168.0058, 331.0669, 149.9954, 313.0571	Glycogallin	[29]
7	7.50	C_7_H_6_O_3_	137.02501	137.02442	4.4	13522	93.0367, 65.0491	Salicylic acid	[28]
9	9.09	C_13_H_16_O_9_	315.07245	315.07216	0.9	537030	152.0112, 108.0225, 315.0727	Protocatechuic acid hexoside	[28, 29]
10	12.20	C_7_H_6_O_4_	153.01971	153.01933	2.5	2950	108.0223, 109.0300, 91.0204	Protocatechuic acid	[28]
11	13.81	C_13_H_16_O_8_	299.07735	299.07724	0.4	96826	137.0242, 93.0356	Hydroxybenzoic acid hexoside	[29]
13	15.47	C_16_H_18_O_8_	337.09287	337.09289	−0.1	1421688	163.0398, 119.0505, 191.0556	5-p-Coumaroylquinic acid	[28, 29]
19	19.57	C_19_H_26_O_13_	461.12986	461.13006	−0.4	5897	429.1068, 167.0325, 233.0686, 461.1565	Saccharumoside C/D	[28]
Flavonoids									
15	16.24	C_27_H_30_O_17_	625.14107	625.14102	0.1	67554	625.1455, 299.0190, 462.0815, 301.0347	Quercetin 3,4'-diglucoside	[30]
18	18.45	C_33_H_40_O_20_	755.20474	755.20402	1	373712	593.1535, 285.0401, 755.2099	Faralateroside	[31]
22	20.35	C_15_H_14_O_6_	289.07167	289.07176	−0.3	2184331	123.0452, 109.0300, 203.0709, 289.0716	(Epi)catechin	[28, 32]
23	22.14	C_21_H_20_O_13_	479.08271	479.08311	−0.8	14584	299.0196, 271.0245, 231.0277, 316.0199	Myricetin-3-galactoside	[32]
24	22.15	C_45_H_38_O_18_	865.19986	865.19854	1.5	926208	865.2097, 287.0562, 289.0718, 577.1395	Procyanidin C	[33]
25	23.08	C_15_H_10_O_8_	317.02998	317.03029	−1	9019	151.0027, 190.9976, 109.0294, 163.0024	Myricetin	[32]
26	26.79	C_27_H_30_O_16_	609.1466	609.14611	0.8	3262275	300.0272, 301.0353, 609.1492, 271.0249	Rutin (Quercetin-3-rutinoside)	[28, 34]
27	28.79	C_21_H_20_O_12_	463.08798	463.0882	−0.5	2248624	300.0364, 301.0350, 271.0237, 463.0890	Isoquercetin	[30, 35]
28	30.30	C_27_H_30_O_15_	593.15172	593.15119	0.9	3946771	593.1530, 285.0393, 284.0316, 255.0293	Kaempferol-3-O-β-D-rutinoside^a^	[28, 34]
29	30.79	C_28_H_32_O_16_	623.16206	623.16176	0.5	18518	623.1663, 315.0508, 314.0448, 299.0205	Isorhamnetin-3-rutinoside	[28]
30	31.14	C_23_H_22_O_13_	505.09892	505.09876	0.3	89414	505.1003, 300.0264, 301.0348, 271.0236	Quercetin 3-(6-O-acetyl-beta-glucoside)	[36]
31	31.14	C_24_H_22_O_15_	549.08848	549.08859	−0.2	55297	505.1016, 300.0281, 301.0359, 271.0253	Quercetin-3-(6-malonyl)-glucoside	[37]
32	31.55	C_20_H_18_O_11_	433.07735	433.07764	−0.7	242264	300.0284, 271.0257, 433.0791, 301.0359	Quercetin-3-D-xyloside	[35]
33	32.55	C_21_H_20_O_11_	447.09323	447.09329	−0.1	2580398	447.0949, 284.0331, 255.0300, 227.0350	Kaempferol-O-glucoside	[28, 32]
35	34.48	C_20_H_18_O_10_	417.08248	417.08272	−0.6	123420	284.0323, 255.0295, 417.0837, 227.0343	Kaempferol-O-arabinoside	[35]
36	35.30	C_24_H_22_O_14_	533.09384	533.09368	0.3	314360	285.0406, 284.0333, 489.1061, 255.0303	Kaempferol-(malonyl)-glycoside	[38]
37	35.30	C_23_H_22_O_12_	489.10399	489.10385	0.3	752145	285.0405, 284.0332, 489.1052, 255.0301	Kaempferol-O-(acetyl)-glucoside	[36]
38	36.33	C_21_H_20_O_10_	431.09809	431.09837	−0.6	684742	285.0401, 284.0329, 255.0295, 431.0998	Kaempferol-O-rhamnoside	[34, 35]
39	40.46	C_15_H_10_O_7_	301.03555	301.03538	0.6	382921	151.0038, 301.0359, 178.9986, 121.0298	Quercetin^a^	[28, 30, 32]
42	43.42	C_22_H_18_O_10_	441.08175	441.08272	−2.2	56589	221.0086, 441.0837, 193.0133, 177.9900	(Epi)catechin-3-O-gallate	[39]
43	43.69	C_15_H_10_O_6_	285.04049	285.04046	0.1	647679	285.0409, 187.0403, 185.0609, 239.0342	Kaempferol^a^	[28, 32]
44	43.90	C_16_H_12_O_7_	315.05075	315.05103	−0.9	9008	300.0275, 315.0511, 151.0025, 271.0225	Isorhamnetin	[40]
Coumarins									
12	14.28	C_16_H_18_O_10_	369.08273	369.08272	0	971835	192.0058, 207.0293, 369.0825	Fraxin	[41]
14	15.94	C_10_H_8_O_4_	191.03517	191.03498	1	187693	176.0108, 148.0161, 104.0277, 191.0336	Scopoletin^a^	[42]
16	17.24	C_11_H_10_O_5_	221.0456	221.04555	0.2	246358	190.9986, 163.0034, 206.0220, 135.0087	Isofraxidin	[43]
21	20.04	C_10_H_8_O_5_	207.03002	207.0299	0.6	9398	192.0041, 164.0101, 136.0166, 108.0213	Fraxetin	[41]
Fatty acids									
40	41.00	C_18_H_32_O_5_	327.2175	327.2177	−0.6	129419	327.2170, 211.1328, 229.1429, 171.1020	Trihydroxy octadecadienoic acid	[28]
41	42.35	C_18_H_34_O_5_	329.23301	329.23335	−1	112949	329.2333, 211.1331, 229.1442, 171.1020	Trihydroxy octadecenoic acid	[28]
46	52.27	C_18_H_32_O_3_	295.22767	295.22787	−0.7	186462	295.2280, 277.2175, 195.1386	Hydroxy octadecadienoic acid	[28]
47	57.08	C_18_H_30_O_2_	277.21703	277.2173	−1	5962	277.2152, 277.1823, 147.0812, 233.1891	Linolenic acid	[28]
Other compounds									
1	2.63	C_6_H_12_O_7_	195.05093	195.05103	−0.5	108318	75.0103, 195.0513, 129.0187	Gluconic acid	[29]
5	4.35	C_9_H_12_N_2_O_6_	243.06226	243.06226	0	11041	110.0261, 111.0120, 200.0524, 82.0352	Uridine	[44]
8	7.59	C_9_H_11_NO_2_	164.07189	164.0717	1.2	7877	103.0543, 147.0473, 164.0704	Phenylalanine	[44]
17	17.57	C_19_H_30_O_8_	385.18645	385.18679	−0.9	23312	153.0916, 205.1228, 385.1874, 223.1331	Roseoside	[29]
20	20.03	C_9_H_8_O_4_	179.03492	179.03498	−0.3	5767	135.0440, 134.0368, 179.0343, 117.0382	Caffeic acid	[45]
45	48.02	C_34_H_60_O_16_	723.3803	723.38086	−0.8	34616	397.1347, 279.2326, 415.1457, 723.3885	Gingerglycolipid B	[28]

^a^Compared with an authentic standard.

### 3.2. The total flavonoid content and individual compound contents of *Chimonanthus nitens* Oliv. leaves

The total flavonoid contents of the CN leaves is shown in Table 2, and the standard curve has a relatively high correlation coefficient (> 0.995) in the linearity range for rutin. Meanwhile, four typical compounds were analyzed in this study and identified and quantified by HPLC—DAD followed by comparisons of their retention time to known amounts of authentic standards. Standard curves were fitted for each compound and proved to have high correlation coefficients (> 0.9995). The results are summarized in Table 2. Combined with the “Ion intensity” data in Table 1, the results also suggested that kaempferol-3-O-β-D-rutinoside (5.0366 mg/g DW) might be the most abundant bioactive component in the CN leaves.

**Table 2:** The total flavonoid contents (TFC) and individual compound contents of *Chimonanthus nitens* Oliv. leaves.

Name	Retention time (min)	Standard curve	R^2^	Liner range (μg/mL)	Content in CN leaves (mg/g DW)
Total flavonoid contents (rutin equivalent)	-	y = 0.0143x + 0.0176	0.9961	10–60	60.73 ± 1.27^a^
Scopoletin	28.79	y = 78031x − 2905.6	1	0.880–8.80	0.0825 ± 0.0016^a^
Kaempferol-3-O-β-D-rutinoside	43.11	y = 23058x − 2781.2	1	4–40	5.0366 ± 0.1655^a^
Quercetin	48.64	y = 35625x − 1594.7	0.9997	0.584–5.84	0.0365 ± 0.0023^a^
Kaempferol	53.82	y = 56771x − 467.2	0.9999	0.448–4.48	0.0438 ± 0.0017^a^

^a^Data were presented as mean ± SD. (n = 3 for TFC or n = 6 for individual compounds)

### 3.3. Anti-inflammatory effects of *Chimonanthus nitens* Oliv. leaf extracts

#### 3.3.1 Survival analysis

Survival rates of zebrafish were examined by counting the number of dead larvae. The results showed that fish mortality did not vary with the doses of CN leaf extracts or LPS in a dose-dependent manner.

For the groups only treated with extracts (0, 5, 10, 25 μg/mL) or DMSO (0.1%, v/v), all of the groups had no mortality rates (0%) at 6 hpi and had lower mortality rates at 24 hpi (< 1%). DEX is an anti-inflammatory drug and a common positive control for anti-inflammatory experiments [20, 46, 47], it should be less toxic and anti-inflammatory at a reasonable dose. The group treated with DEX (5 μg/mL), as the positive control, had no mortality rate (0%). The effect of LPS doses on the fish mortality was also evaluated. For the groups injected with LPS (up to 5 ng for per injection), results showed lower mortality rates (< 2%) within 24 hpi. Zebrafish showed a strong tolerance against LPS in our study [20]. For the groups injected with LPS (5 ng) and then treated with extracts (0, 5, 10, 20 μg/mL) or DEX (5 μg/mL), mortality rates were less than 1% within 24 hpi. Based on the results of survival analysis, the experimental doses were determined.

#### 3.3.2 Inflammatory cell migration analysis

**Fig 2:**
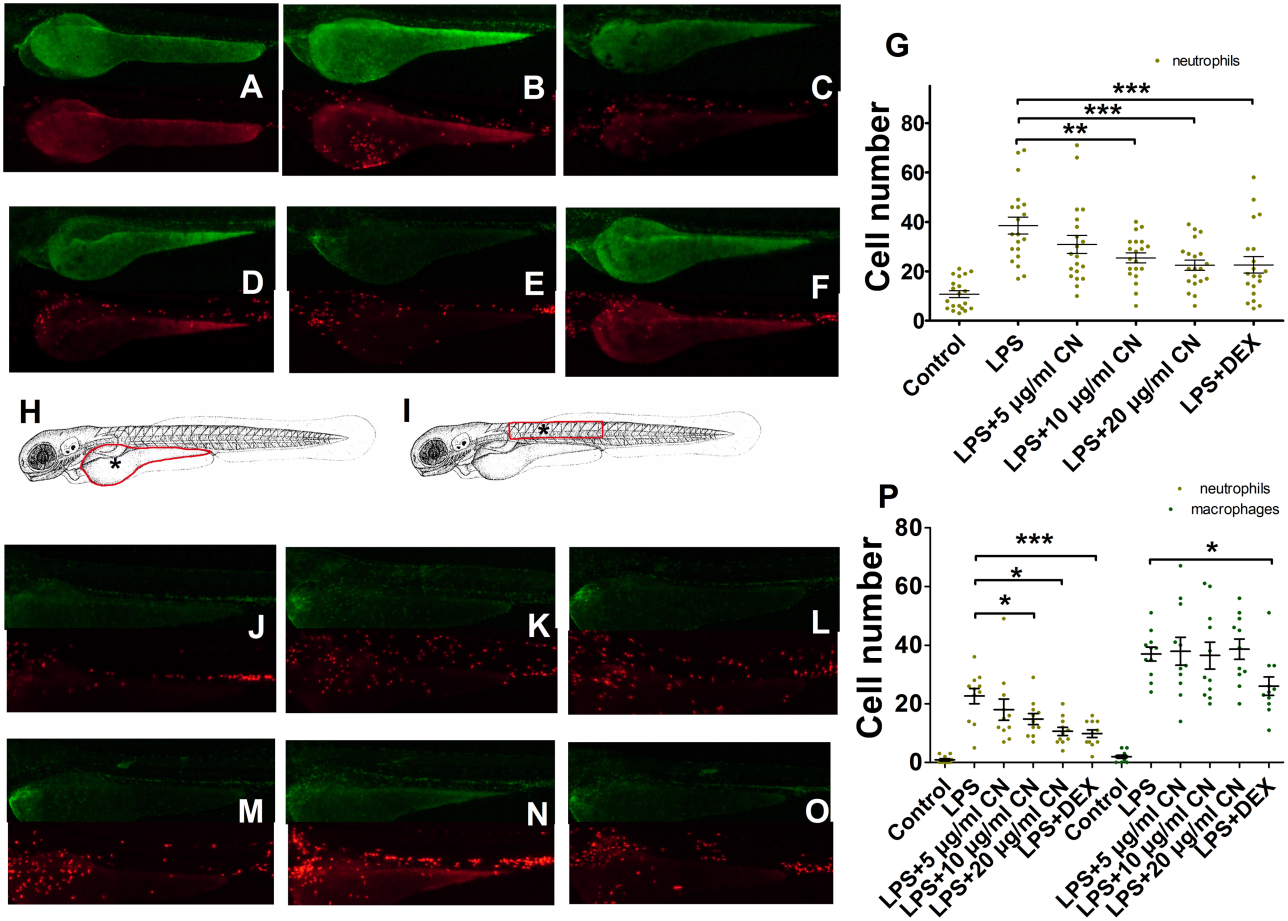
Macrophages and neutrophils migrated to the injured area were count. A-F, images show the macrophages and neutrophils migrated to the yolk microinjected by LPS at 6 hpi. G, neutrophils count in yolk at 6 hpi (n = 20). H and I, microinjection site were marked by * and cell count area were marked by red circle. J-O, images show the macrophages and neutrophils migrated to the somite muscle microinjected by LPS at 6 hpi. P, macrophages and neutrophils count in somite muscle at 6 hpi (n = 11). Data were shown as mean ± S.E. For G and P, * indicates p<0.05, ** is p<0.01, *** is p<0.001.

The transgenic zebrafish were microinjected with LPS, then treated with different concentrations of CN leaf extracts or DEX. Macrophages and neutrophils that migrated to the injured area were counted at 6 hpi. Since the green fluorescence labeled cells were difficult to observe in yolk because of the background color, the number of macrophages which migrated to the yolk was not counted. As shown in Fig 2A–2G, in the injured area of zebrafish injected with LPS in yolk, the number of neutrophils was up to 38.50 ± 3.41 (3.58-fold to the control group). When zebrafish individuals injected with LPS were treated with 5, 10, 20 μg/mL CN extracts or 5 μg/mL DEX, the number of neutrophils was decreased significantly, 30.85 ± 3.65 (p = 0.073), 25.40 ± 2.01 (p < 0.01), 22.45 ± 2.08 (p < 0.001) and 22.60 ± 3.34 (p < 0.001), respectively. As shown in Fig 2J–2P, after LPS was microinjected into zebrafish somite muscle for 6 h, the number of neutrophils in wound area was up to 22.64 ± 1.94 (24.88-fold to the control group), and the number of macrophages was up to 36.91 ± 1.76 (18.46-fold to the control group). Similarly, when zebrafish injected with LPS were treated with 5, 10, 20 μg/mL CN extracts or 5 μg/mL DEX, a significant dose-dependent decrease in the number of neutrophils was observed, 18.00 ± 2.67 (p = 0.165), 14.82 ± 1.40 (p < 0.05), 10.64 ± 1.04 (p < 0.05) and 9.82 ± 0.98 (p < 0.001), respectively. However, the number of macrophages did not show a significant change in the injured area of somite muscle, except the DEX treated group (p < 0.05). It could be concluded that the CN extracts had no significant effect on macrophages migration, but had an impact on neutrophils.

#### 3.3.3 Inflammatory cytokine mRNA expression analysis

**Fig 3:**
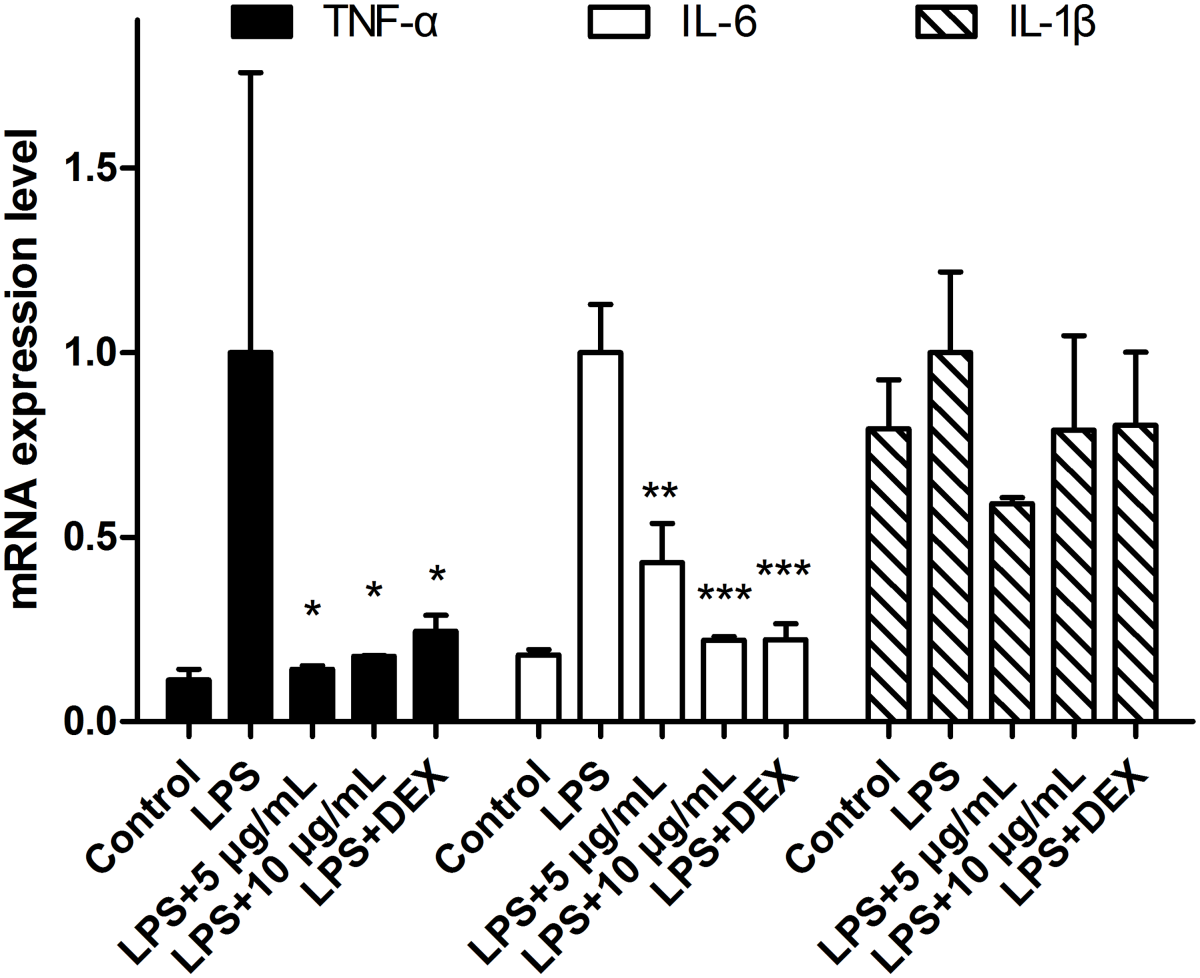
The effect of CN extract on mRNA expression of TNF-α, IL-6 and IL-1β. Data were shown as mean ± S.E. (n = 3). * indicates p<0.05, ** is p<0.01, *** is p<0.001.

To elucidate the inhibitory effect of CN leaf extracts on cytokine production in LPS stimulated zebrafish, the mRNA expression of several proinflammatory cytokines (TNF-α, IL-6 and IL-1β) at 24 hpi was examined by q-PCR. The mRNA expressions of all three cytokines were compared with the expression level of β-actin. Extracts were used in this study at concentrations of 5 and 10 μg/mL. As shown in Fig 3, compared to the LPS group, the mRNA expressions of TNF-α, IL-6 and IL-1β were up to 13.07-, 5.56- and 1.30-fold, respectively. For the LPS stimulated zebrafish treated with the CN leaf extracts or DEX, the mRNA expressions of TNF-α, IL-6 and IL-1β were also evaluated. The CN extracts had no significant effect on the expression of IL-1β mRNA. For TNF-α, the CN leaf extracts reduced the production of mRNA at concentrations of 5 μg/mL (↓90.4%, p < 0.05) and 10 μg/mL (↓88.0%, p < 0.05). In addition, extracts significantly reduced IL-6 mRNA expression at concentrations of 5 μg/mL (↓56.9%, p < 0.01) and 10 μg/mL (↓78.0%, p < 0.001) in a dose-dependent manner. It could be concluded that the CN leaf extracts had no effect on the expression of IL-1β in LPS stimulated zebrafish model, but had an effect on the expression of TNF-α and IL-6.

#### 3.3.4 RAW 264.7 cell viability analysis

**Fig 4:**
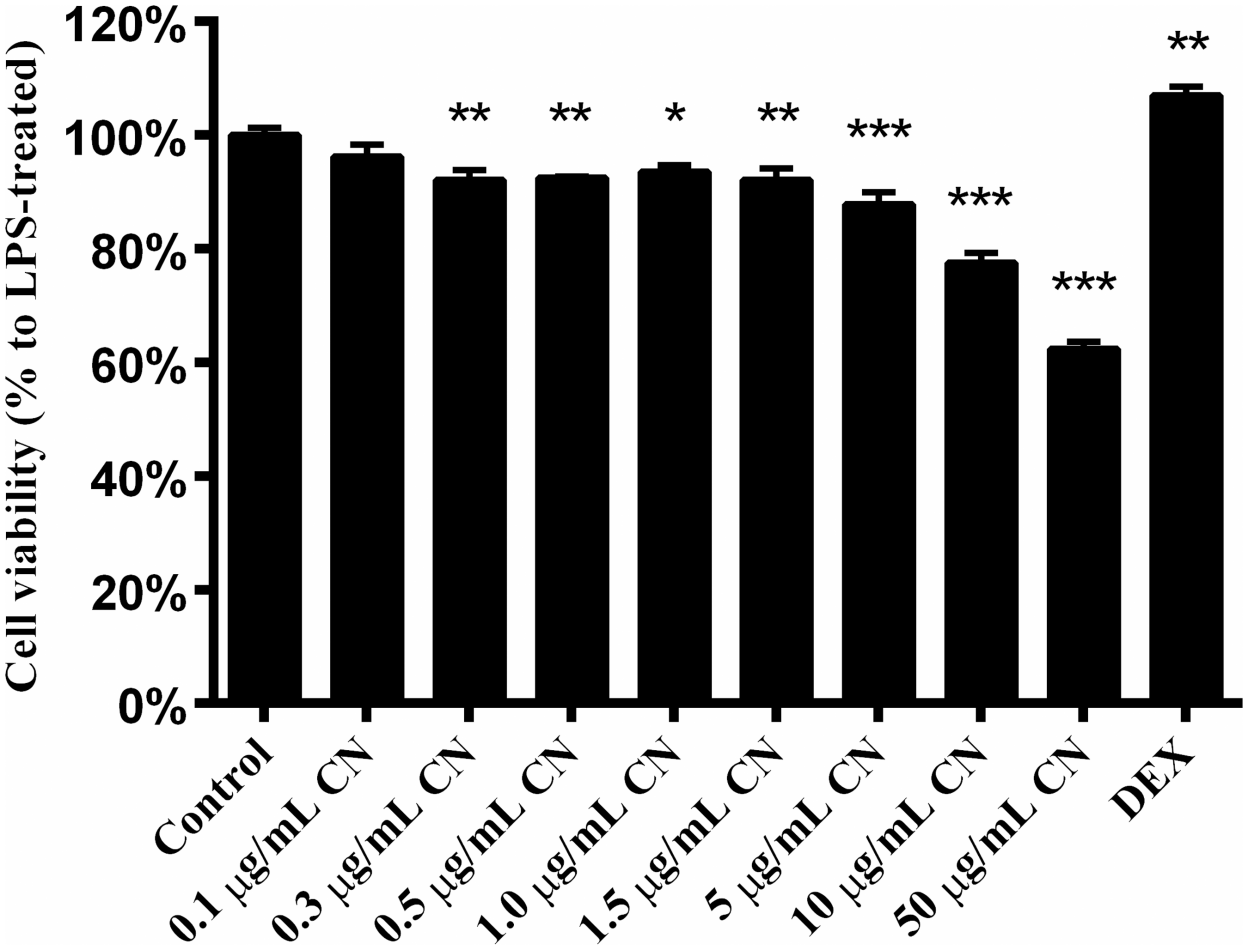
The effect of CN extract on cell viability. Data were shown as mean ± S.E. (n = 3). * indicates p<0.05, ** is p<0.01, *** is p<0.001.

RAW 264.7 macrophages were incubated with CN leaf extracts for 24 h. Cell viability was analyzed using MTT assay. The result showed little or no effect on cell viability relative to the control group at concentrations lower than 1.5 μg/ml (Fig 4). However, the cells treated with the higher concentration (50 μg/ml) of CN leaf extracts showed a reduced viability of 62.35%. Based on this result, non-toxic concentrations of CN leaf extracts (0.1 and 1.0 μg/ml) were used in the subsequent experiments.

#### 3.3.5 Inflammatory cytokine expression level analysis

**Fig 5:**
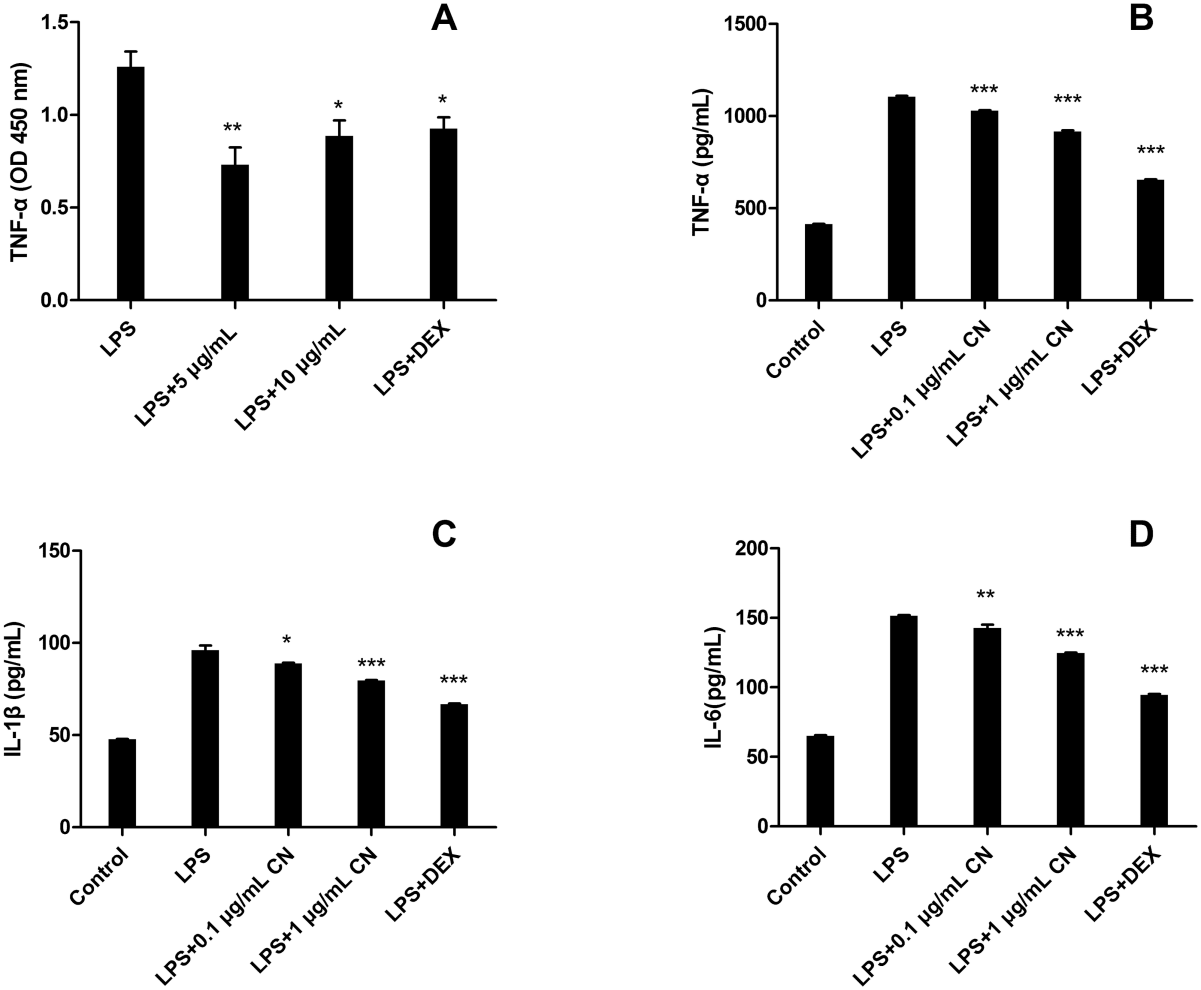
Inhibitory effect of CN leaf extracts on the production of pro-inflammatory cytokines in zebrafish (A) or RAW 264.7 macrophages (B, C, D). Data were shown as mean ± S.E. (n = 3). * indicates p<0.05, ** is p<0.01, *** is p<0.001.

To evaluate the the inhibitory effect of CN leaf extracts on proinflammatory cytokine production, the production level of cytokines (TNF-α, IL-6 and IL-1β) was examined by ELISA. Extracts were used in this study at concentrations of 5 and 10 μg/mL for zebrafish model. As shown in Fig 5A, compared to the control group, extracts significantly reduced TNF-α production at concentrations of 5 μg/mL (↓41.9%, p < 0.005) and 10 μg/mL (↓29.6%, p < 0.01) in LPS-stimulated zebrafish. The results are roughly consistent with the TNF-α mRNA expression analysis.

The inhibitory effect of CN leaf extracts on proinflammatory cytokine production in RAW 264.7 cells was also examined. As shown in Fig 5B–5D, after LPS induction, the expression levels of three inflammatory factors were increased to 2.66-fold (1103.50 pg/mL for TNF-α), 2.05-fold (98.07 pg/mL for IL-1β) and 2.33-fold (151.38 pg/mL for IL-6). Compared to the LPS group, extracts significantly reduced TNF-α, IL-6 and IL-1β production in LPS-stimulated RAW 264.7 cells, in dose-dependent manners. When LPS-induced cells were treated with the 1.0 μg/ml of the extracts, the production of the inflammatory factors was significantly inhibited to 918.88 pg/mL (for TNF-α, p<0.001), 79.74 pg/mL (for IL-1β, p<0.001) and 124.72 pg/mL (for IL-6, p<0.001).

## Discussion

Inflammation has been shown to delay healing and lead to increased scar formation. Meanwhile, chronic inflammation also contributes to cancer and other human diseases [48, 49]. Therefore, human body requires suitable regulation of inflammatory responses. However, nonsteroidal anti-inflammatory drugs that have been used to suppress inflammation may cause gastrointestinal bleeding and ulcers. Growing evidence suggests important roles of dietary factors in preserving health and also suggests that natural products can decrease inflammation. In this study, we characterized the profile of ethanol extracts from *Chimonanthus nitens* Oliv. leaf by UPLC-QTOF-MS/MS and HPLC-DAD and then investigated the protective effects of CN extracts in zebrafish and RAW 264.7 inflammation models.

Many studies have studied the anti-inflammatory effects of the active extracts or compounds on the expression of pro-inflammatory factors (such as TNF-α, IL-6, IL-1β, IL-8, NF-κB and COX-2) in the zebrafish model only using PCR analysis [27, 50–55], but without further Western Blot or ELISA analysis, the reason might be the underdevelopment of the antibody industry for zebrafish. However, not all the transcripts are translated into proteins. The q-PCR results should be validated with ELISA to show the differences and immunoblotting. In the present study, TNF-α antibody for zebrafish was used to explore the effect of the CN leaf extracts on the expression of TNF-α in zebrafish model. In addition, LPS-stimulated RAW 264.7 cell model was also used for ELISA to explore the expressions of TNF-α, IL-6, IL-1β in order to better understand the anti-inflammatory property of CN leaf extracts and support the conclusions.

Injecting LPS produces both trauma and harmful stimuli that can cause inflammation, and can be considered as an acute inflammation. When LPS was injected into the body, the neutrophils are at first attracted to the wound site, then proliferate. The first wave of neutrophils stimulates the appearance of the macrophages, which will then engulf the aged neutrophils [49]. Both macrophages and neutrophils are stimulated by LPS and release cytokines (TNF-α, IL-6 and IL-1β) in tissue repair to amplify inflammatory response [49]. The migration process of inflammatory cells is closely related to the expression of pro-inflammatory cytokines. Subsequently, the number of macrophages and neutrophils increased at the injured site, accompanied with further increase of pro-inflammatory cytokines. The LPS-stimulated zebrafish model is a model that applied in many studies to evaluate subject’s anti-inflammatory effects [20, 55]. Macrophages can be recognized in zebrafish as early as the 15 h post fertilization (hpf) [56]. Then the neutrophils can been found in the trunk and tail by 48 hpf [57]. Therefore macrophage and neutrophil can be observed in zebrafish larvae at 3 dpf, and are highly attracted to local infections [57]. In the present study, CN leaf extracts (5, 10 and 20 μg/mL) suppressed LPS-stimulated recruitment of neutrophils in zebrafish in a dose-dependent manner. TNF is produced by a broad variety of cell types including macrophages in response to LPS, other bacterial products, and IL-1 [58]. TNF-α can stimulate phagocytosis on macrophages and help neutrophils migrate to the injured site. In this study, CN leaf extracts inhibited LPS-induced TNF-α mRNA and protein levels in zebrafish and RAW 264.7 cells. IL-6 is secreted by macrophages and T cells to stimulate immune response and create pro-inflammatory responses in macrophages [59]. CN leaf extracts treatment significantly reduced LPS-induced IL-6 mRNA expression in zebrafish and IL-6 protein levels in RAW 264.7 cells in dose-dependent manners. IL-1β is also an important mediator of the inflammatory response, and can induce the production of COX-2. CN leaf extracts treatment reduced IL-1β production in LPS-stimulated RAW 264.7 cells in a dose-dependent manner. The inhibitory ability of the extracts in the zebrafish model and the cell model is not exactly the same, which may be due to different complexity of the functional system of living organisms and cell lines. These zebrafish experiment results suggest that the CN extracts may inhibit the mRNA expression of inflammatory factors TNF-α and IL-6 and thus inhibit the inflammation by reducing the number of neutrophils migrating to the injured site. The results also indicate that the compounds in ethanol extracts of CN leaf may serve as potential anti-inflammatory phytochemicals.

Forty seven chemical compounds were identified from the CN leaf extracts in this work, and the presence of most of the compounds screened has not previously been studied in *Chimonanthus nitens* Oliv. leaf, based on our literature survey. Numerous recent phytochemical studies have increased our understanding of the pharmacological action of these compounds in the CN leaf extracts. Many studies have reported the function of flavonoids, such as targeting obesity and inflammation, antioxidant and anticancer activities [60–63]. CN leaf extracts contain a high amount of flavonoids, especially flavonols. Consequently, it is possible that the flavonoid constituents present in CN leaf extracts could contribute to the antioxidative and anti-inflammatory activity of CN leaf. Quercetin (identified compound **39**) has been found to display the anti-inflammatory activity in LPS-stimulated RAW 264.7 cells by controlling pro-inflammatory cytokines (TNF-α, IL-6 and IL-1β) and NO/iNOS [64]. It was also reported that isoquercetin (**27**) could act as an inhibitor on the formation of the carrageenan-induced hind paw edema, PGE2-induced hind paw edema and TPA-induced ear edema models [65]. Kaempferol-O-rhamnoside (**38**) was found to inhibit superoxide anion generation, while (Epi)catechin (**22**) and Kaempferol-O-glucoside (**33**) could inhibit neutrophil elastase release[66]. Meanwhile, coumarins and their derivatives have shown anti-tumor, anti-viral, anti-inflammatory and antioxidant effects, as well as anti-microbial properties [67, 68]. Fraxin (**12**) was revealed to inhibit the mRNA expression of iNOS and COX-2 in HaCaT cells (extracted from the roots of *Ulmus macrocarpa*) [69] and have significant anti-inflammatory effect in acetic acid-induced vascular permeability experiment in mice (extracted from *Vaccinium vitis-idaea*) [70]. Scopoletin (**14**) was reported to reduce the overproduction of PGE2 and TNF-α in croton oil-treated mouse ears [71]. The alleviating effect of CN extracts is probably also attributed to their coumarins composition. Since the extract contains a large number of different types of compounds, further isolation work with anti-inflammatory properties still needs to be undertaken.

## Conclusions

The present study demonstrated the profiles of the ethanol extracts of *Chimonanthus nitens* Oliv. leaves and evaluated its anti-inflammatory properties. The presence of many compounds, including coumarins and flavonoids, was reported for the first time in the CN leaves. The total flavonoid contents of the CN leaves and four typical compounds in CN leaves were measured. Additionally, the anti-inflammatory effects of CN leaf extracts were demonstrated both *ex vivo* and *in vivo* experiments. The CN extracts showed high activities to inhibit the recruitment of neutrophils and the expressions of TNF-α and IL-6 in LPS-stimulated zebrafish and RAW 264.7 cells. The extracts also inhibit the expression of IL-1β in the cells. Given the complexity of the components in the extract, further work is needed to screen and understand the anti-inflammatory ability of individual components in the CN extracts.
